# Impedance of the Grape Berry Cuticle as a Novel Phenotypic Trait to Estimate Resistance to *Botrytis Cinerea*

**DOI:** 10.3390/s150612498

**Published:** 2015-05-27

**Authors:** Katja Herzog, Rolf Wind, Reinhard Töpfer

**Affiliations:** Julius Kühn-Institut-Federal Research Centre of Cultivated Plants, Institute for Grapevine Breeding Geilweilerhof, Siebeldingen 76833, Germany; E-Mails: rolf.wind@kdwelt.de (R.W.); reinhard.toepfer@jki.bund.de (R.T.)

**Keywords:** sensor development, phenotyping, grapevine breeding, berry skin, objective data, bunch compactness, *Vitis vinifera*

## Abstract

Warm and moist weather conditions during berry ripening provoke *Botrytis cinerea* (*B. cinerea*) causing notable bunch rot on susceptible grapevines with the effect of reduced yield and wine quality. Resistance donors of genetic loci to increase *B. cinerea* resistance are widely unknown. Promising traits of resistance are represented by physical features like the thickness and permeability of the grape berry cuticle. Sensor-based phenotyping methods or genetic markers are rare for such traits. In the present study, the simple-to-handle I*-*sensor was developed. The sensor enables the fast and reliable measurement of electrical impedance of the grape berry cuticles and its epicuticular waxes (CW). Statistical experiments revealed highly significant correlations between relative impedance of CW and the resistance of grapevines to *B. cinerea*. Thus, the relative impedance *Z_rel_* of CW was identified as the most important phenotypic factor with regard to the prediction of grapevine resistance to *B. cinerea*. An ordinal logistic regression analysis revealed a *R^2^*_McFadden_ of 0.37 and confirmed the application of *Z_rel_* of CW for the prediction of bunch infection and in this way as novel phenotyping trait. Applying the I-sensor, a preliminary QTL region was identified indicating that the novel phenotypic trait is as well a valuable tool for genetic analyses.

## 1. Introduction

**Figure 1:**
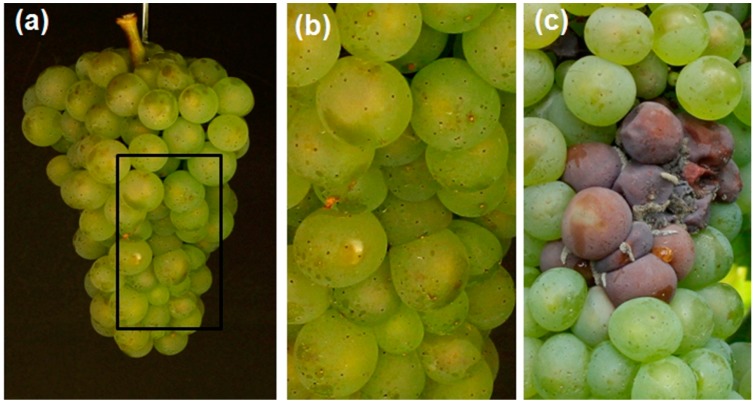
The compactness of grapevine bunches as one major reason for the susceptibility of grapevines to *B. cinerea*. Compact bunches of the susceptible grapevine cultivar ‘Riesling’ (**a**); showing regions where berries are very dense (**b**); and often the growth of *B. cinerea* begins in these regions as result of damaged berries (**c**).

Grey mold is a plant disease caused by the ubiquitous fungus widely known as Botrytis that affects more than 200 plant species [1]. The necrotrophic pathogen and filamentous fungus *Botrytis cinerea* Pers., abbreviation *B. cinerea*, is the anamorph of the ascomycete *Botryotinia fuckeliana* Whetzel. On grapevine (*Vitis vinifera* L.) it causes one of the most serious diseases, the bunch rot. This disease can drastically reduce both the yield at harvest time and wine quality, and can be controlled by specific canopy management, *i.e.*, the reduction of foliage around grape bunches (literature overview is given by Molitor, *et al.* [2], Broome, *et al.* [3]) permitting a faster drying of grape bunches. In years with persistent rain during the ripening period, the effectiveness of canopy management is limited and expensive fungicide applications [4] or, at the expenses of quality, a premature harvest is necessary in order to keep yield losses at a minimum. Furthermore, bunch rot can be observed especially on grapevines with compact bunch architecture [2,5,6,7,8], as illustrated in Figure 1, and occurrence of the disease is most notable in years with moist and warm weather conditions during ripening of the grape berry [3,9,10]. With regard to that, in grapevine breeding programs, seedlings will be selected with convenient physical properties, e.g., loose bunch architecture and small berries.

**Figure 2:**
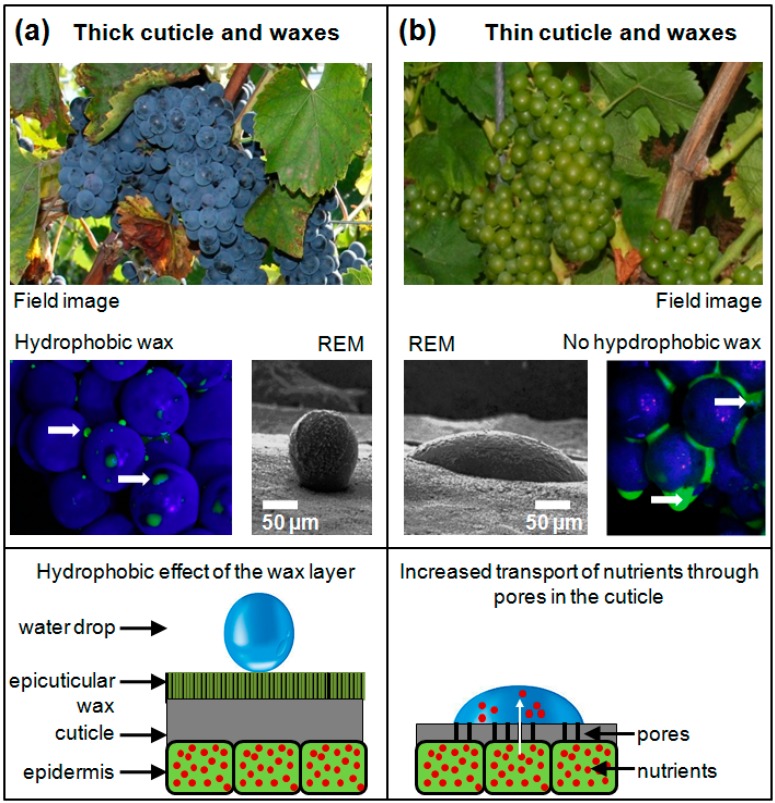
Function of the cuticle and epicuticular waxes as physical barriers. (**a**) As an example of grape berries with a thick cuticle and waxes, the grapevine accessions Seibel 182 from the genetic repository in Siebeldingen was used. The hydrophobic characteristic of epicuticular waxes (wax layer) permits fast drying of berry surfaces; (**b**) As an example of grape berries with thin cuticle and waxes the grapevine cultivar ‘Morio Muskat’ is shown. The absence of epicuticular waxes results in an accumulation of water between berries. Fluorescein (yellow-green) stained water (arrows) as well as Raster Electron Microscope (REM) were used to illustrating the effect.

Besides compactness, different berry skin features seem to influence the susceptibility of grapevines towards *B. cinerea* infection, *i.e.*, the biochemical composition [10,11,12,13], the ripening stage [10,12] and the morphology of the berry skin [11,14]. Especially, the cuticle and its epicuticular waxes are described as important berry skin features regarding the susceptibility of berries toward bunch rot [11,15,16]. In this context, warm temperatures, high air humidity and water on the berry surface are known as major reasons for the incidence of microscopic cracks in the cuticle membrane of berries [15,16]. These, in turn, play a critical role in the susceptibility of grape berry against *B. cinerea*, since they impair the function of the cuticle as a barrier for pathogen defense and permit an increased water uptake into the berries [16]. The cuticle forms the outer surface of leaf, fruit and primary-shoot epidermal cell walls [17,18] and additionally serves as a regulator of molecular diffusion [19]. It consists of intra- and epicuticular waxes, which build up a hydrophobic berry surface. That, in turn, achieves a faster drying of berries/bunches, which is described as an important factor in reducing the susceptibility of grapevines to *B. cinerea*. It is also known that polar pores appear through high air humidity and warm conditions resulting in an increased water permeability of this pores [20,21]. Polar pores in return facilitate the diffusion of organic substrates (*i.e.*, sugar, nutrients) to the berry surface and promoting growth of *B. cinerea*. Figure 2 illustrates the impact of the thickness of cuticle and epicuticular waxes on: (1) transport of nutrients to the berry surface; (2) hydrophobic property on the berry surface; and (3) accumulation of water between berries of compact bunches.

**Figure 3:**
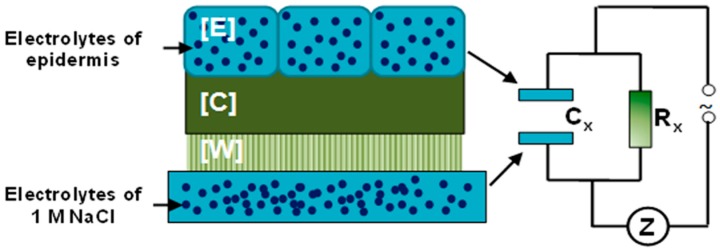
Schematic distribution of electrolytes and the physical principle of impedance measurements. The cuticle [C] and the epicuticular wax layer [W] located between two electrically conducting surfaces, the epidermis [E] and NaCl solution. The impedance *Z* is the sum of the imaginary resistor (C_x_ (thickness of cuticle, wax layer and air)) and the real resistor (R_x_ (permeability of [C] and [W]).

The current bottleneck in phenotyping physical characteristics of berries is the lack of an easy and reliable method. The phenotyping methods described in previous studies (e.g., phenotyping the thickness of the epidermal layer or number of pores or existence of microscopic cracks) are laborious and very time consuming [11,15,16]. Hence, the acquisition of these traits is not feasible for common grapevine breeding programs where hundreds of different genotypes need to be evaluated. Therefore, the development of sensor-based methods is required in order to phenotype the grape berry skin and the influence to the bunch rot susceptibility of grapevines. The method should be rapid, permitting the generation of objective phenotypic data from a large number of samples. With regard to this, the measurement of electrical impedance *Z* was chosen to characterize electrical behavior of the berry cuticle (C) and epicuticular waxes (W) as a novel phenotypic trait as well as an indicator for thickness and permeability of C and W (Figure 3).

The first aim of the present study was the development and validation of a sensor to determine impedance of the cuticle of grape berries with epicuticular waxes (*Z_rel_* of CW) and the cuticle without epicuticular waxes (*Z_rel_* of C). Secondly, both physical parameters were measured from different grapevine cultivars to investigate if there is any relationship between impedance and the susceptibility of grape berry toward *B. cinerea* infection and bunch rot, respectively. Finally, as a test of the application, the impedance was measured in a F1 progeny (crossing of GF.GA.47-42 x 'Villard Blanc') in order to test the novel phenotypic data for its utilization in QTL (Quantitative-Trait-Loci) analysis.

## 2. Experimental Section

### 2.1. Plant Material and Sampling

As plant material, 41 different genotypes (including traditional cultivars, breeding material and cultivars from the genetic repository planted at the experimental vineyard of Geilweilerhof located in Siebeldingen, Germany ((N 49°21.747, E 8°04.678), overview in Table S1) were used for method validation. In addition, 144 genotypes of a F1 progeny of the crossing GF.GA-47-42 (crossing of 'Bacchus Weiss' x 'Seyval') x 'Villard Blanc' (crossing of 'Seibel 6468' x 'Subereux') were used for phenotyping and QTL analysis. All investigated grapevines are planted in North-South orientation in the vineyards at Geilweilerhof.

Berry ripening for each genotype was monitored weekly by the measurement of sugar content applying a handheld refractometer (VWR^®^ International GmbH, Darmstadt, Germany). Once the sugar level of berries reached approximately 70° Oechsle (*i.e.*, 17.1% Brix), two bunches were sampled (one from the east and the other one from the west side of the plant). Fifteen visually intact berries were randomly sampled per bunch (30 berries per cultivar) by cutting them off carefully at the berry pedicel.

### 2.2. Construction of the I-Sensor

**Figure 4:**
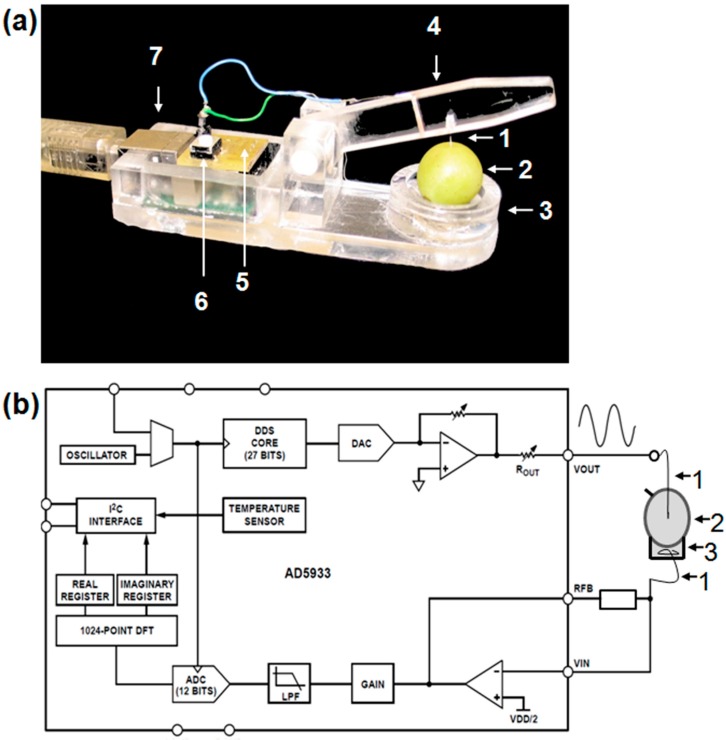
Setup (**a**) and functional block diagram (**b**) of the I-sensor. 1 = sensing electrodes; 2 = grape berry; 3 = skip for NaCl solution; 4 = mobile sensor head; 5 = AD5933 impedance converter; 6 = Button for sensor calibration; and 7 = USB-I^2^C module.

Prototype I-sensor was developed to acquire the electrical impedance of the grape berry cuticle and epicuticular waxes (Figure 4).

The AD5933 high precision impedance converter system (Analog Diveces GmbH, Munich, Germany) fused with a USB-I^2^C module (Devantech Ltd (Robot Electronics, Norfolk, United Kingdom), which provides a complete interface between PC and the I^2^C (*Inter Integrated Circuit*) bus. The USB-I^2^C module uses the FTDI FT232R USB chip. Platinum-Iridium wires with a diameter of 0.3 mm were used as sensing electrodes. For sensor operating, *i.e.*, configuration, calibration, measurement, and result export, a software tool was developed using Embarcadero Delphi Version 3 (Borland^®^, Austin, TX, USA).

### 2.3. Impedance Measurements

For the measurement of the impedance *Z*, single grape berries were placed in the I-sensor (component 3 in Figure 4a) containing a 1 M sodium chloride (NaCl) solution. The mobile sensor head was used to prick the Platinum-Iridium wire (sensing electrode) into the berry. Measurements were conducted at room temperature using an electrical frequency of 2 KHz and 30 KHz. The lower frequency of 2 KHz represents the permeability of the cuticle (C) and cuticle with epicuticular waxes (CW) because it is closely related to the direct current (DC), which is used to determine the threshold voltage. In addition, the higher frequency of 30 KHz represents the thickness of C and CW. The acquisition of *Z* of CW was carried out by measuring visual intact berries twice. Afterwards, the epicuticular waxes were mechanically removed by carefully rubbing using Kimtech-Science^®^ Precision wipes (Kimberly-Clark^®^ Professional, Kimberly-Clark GmbH, Koblenz-Rheinhafen, Germany). The mechanical removal of the wax layer was the most practical, cheapest and fastest way and ensured that the cuticle or other berry skin components were not changed in its chemical or physical properties as it would be the case by using chemicals for eliminating the wax layer. The measurements were repeated to acquire *Z* of C. For both, two different berry positions (lateral and bottom) from the 30 sampled berries were determined, *i.e.*, 60 impedance measurements per genotype and treatment. Subsequently, the berries were bisected and the basis impedance *Z_B_* (impedance of the berry flesh) was determined.

The impedance *Z* at 2 KHz and 30 KHz were used to calculate the relative impedance *Z_rel_,* whereby *d* is the difference between the used electrical frequency (*d* = 28)

(1) $$Zrel=0.5\times (Z_{2KHz}+Z_{30KHz})\times d\times 0.001$$

For further investigations, the median of the 60 *Z_rel_* values (minus *Z_B_*) was calculated for each genotype.

### 2.4. QTL Analysis

The median of the relative impedance was determined from berries of 144 plants of the F1 progeny (crossing of GF.GA-47-42 x 'Villard Blanc'). The population and a first map is described by Zyprian, *et al.* [22] and was extended with additional markers [23,24]. In the present study this extended map was used for QTL analysis. QTL analysis was carried out applying MapQTL^®^ 6.0 (Kyazma^®^, Wageningen, The Netherlands) as described by Fechter, *et al.* [23]. Interval mapping (IM) and permutation test were used to identify preliminary QTLs whose flanking markers were used as co-factors for multiple QTL mapping (MQM).

### 2.5. Reference Evaluations

**Figure 5:**
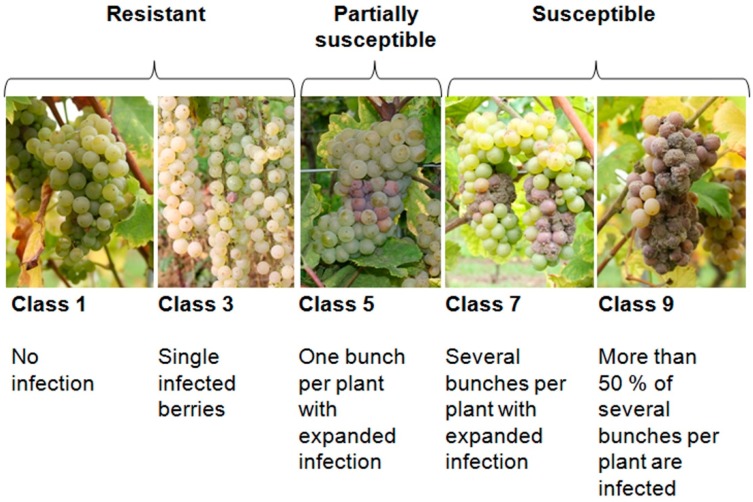
Classification of grapevine susceptibility to *B. cinerea* infection.

The grapevine phenology was evaluated using the BBCH scale (Biologische Bundesanstalt, Bundessortenamt und Chemische Industrie) [25]. It is a commonly used evaluation system to describe predefined stages of plant development. The compactness of grape bunches was classified in parallel to berry sampling by using the OIV (International Organization of Vine and Wine) descriptor 204 [26]. Therefore, the bunch compactness was estimated for all bunches of numerous plants when bunches were sampled for impedance measurements. The susceptibility of genotypes to *B. cinerea* was classified under natural field conditions three weeks after the measurement of impedance. The OIV descriptor 459 was used to create a modified five-class scale (Figure 5).

The aim was the estimation of the risk for *B. cinerea* infection. Genotypes classified in class 3 show only single *B. cinerea* infected berries. These grapevines were denoted as resistant. When several bunches showed many *B. cinerea* infected berries, they were classified as class 3–5, and when the infection expands (example in Figure 1) they were classified as class 5 (*i.e.*, partially infected). This is the beginning of spread of the *B. cinerea* infection, which could be a problem during ripening especially in times of persistent rain. When the infection has expanded on several bunches the grapevines were classified as susceptible. 

It was assumed that genotypes that were classified into: class 1 and 3 are resistant; class 5 are partially susceptible; and class 7 and 9 represent susceptible genotypes.

### 2.6. Statistical Analysis

For method validation, different statistical analyses were conducted using the software SAS^®^ (Statistical Analysis System) Enterprise Guide 4.3 (SAS Institute Inc., Cary, NC, USA). The mean relative impedance *Z_rel_* of investigated berries was compared with *B. cinerea* resistance of the regarding genotype, which had been evaluated in the field. One-way ANOVA analysis, *Pearson* correlation coefficient and *Duncan* multiple range test with the level of significance *α* = 0.05 were conducted. Logistic regression analysis (PROC LOGISTIC) was performed. For the prediction of the probability of *B. cinerea* infection (Five class classification) Maximum Likelihood estimation was used. McFadden’s pseudo coefficient of determination *R^2^* (*R^2^_McFadden_*) was calculated utilizing −2 LOG L (*i.e.*, the log Likelihood of the fitted model) of L0 (constant model) and L1 (constant model and covariates).

(2) $${R^{2}}_{\text{McFadden}}=1-\left(\frac{L_{1}}{L_{0}}\right)$$

## 3. Results and Discussion

### 3.1. Validation the Functionality of the I-Sensor

For the validation of the I-sensor measurements, the impedance was determined 50-times from five visually intact berries of three grapevine cultivars. Table S1 shows the mean impedance for the three cultivars, indicating significant differences between genotypes. The data obtained by the I-sensor proved to be highly reproducible with low standard deviation (SD). Thus, the instrument was usable in a novel phenotyping approach to characterize grape berry cuticle and epicuticular waxes.

### 3.2. Novel Phenotypic Trait as Indicator for Resistance of Grapevines to B. cinerea

The *Pearson* correlation coefficient (Table 1) and significance of correlation were calculated to consider the relation of the mean *Z_rel_* values from 40 grapevine genotypes (Figure S1) with the corresponding class of susceptibility to *B. cinerea*.

**Table 1:** *Pearson* correlation coefficients and significance of correlation of relative impedance *Z_rel_* and bunch compactness determined in comparison to the evaluated *B. cinerea* susceptibility of grapevine cultivars in the field. CW: intact cuticle with epicuticular waxes; C: cuticle without epicuticular waxes; W: epicuticular wax calculated by the substraction of *Z_rel_* of CW and *Z_rel_* of C.

*Z_rel_*	Architecture	Remark	Susceptibility to *B. cinerea*	Significance
CW		Z_rel_ of CW	**−0.67**	<0.0001
C		Z_rel_ C	−0.60	<0.0001
W		Z_rel_ of CW - Z_rel_ of C	−0.53	0.0004
	Bunch compactness		0.40	0.0096

The highest negative correlation was detected between *Z_rel_* of CW (the cuticle of grape berry with epicuticular waxes) and *B. cinerea* susceptibility. This result indicates the importance of both berry skin features with regard to the mechanical protection towards *B. cinerea*. It was observed that genotypes revealing impedance values of CW of 600 or greater show high resistance to *B.cinerea*. In contrast to the literature [2,5,6,7,8], in the present study, the bunch compactness showed only a minor positive correlation to the infection of bunches with *B. cinerea*. This result revealed that the impedance of the berry cuticle and its waxes plays an important role with respect to the susceptibility of investigated cultivars to bunch rot.

To normalize the phenotypic data, the data set was grouped depending on the evaluated grape bunch compactness, *i.e.*, loose, medium or compact bunches. Again, the *Pearson* correlation coefficients and significance of correlations were calculated for each group (Table 2).

**Table 2:** *Pearson* correlation coefficient and significance of correlations for the susceptibility of grapevines to *B. cinerea* and the mean *Z_rel_* of CW, C and W. Grapevine genotypes were grouped according to their bunch compactness, which was evaluated using OIV descriptor 224. The correlation coefficient was rated according to Bühl [27]. N = number of samples; CW: intact cuticle with epicuticular waxes; C: cuticle without epicuticular waxes; W: epicuticular wax.

Bunch Compactness	N	*Z_rel_*	Susceptibility to *B. cinerea*	Significance	Rating of Correlation
loose	10	CW	−0.43	n.s.	Low
		C	−0.22	n.s.	Low
		W	−0.50	n.s.	Low
medium	17	CW	−**0.72**	0.001	**High**
		C	−0.57	0.0173	Medium
		W	−0.61	0.01	Medium
compact	21	CW	−**0.80**	<0.0001	**High**
		C	−**0.83**	<0.0001	**High**
		W	−0.62	0.0027	Medium

It was discovered that the susceptibility to *B. cinerea* is significantly correlated with the impedance *Z_rel_* when the bunch compactness was medium or high. Presumably, the physical property of loose bunches lead to a lower *B. cinerea* infection risk. In loose bunches, the berries do not touch each other. It is considered that the contacts between berries results in violation of the cuticle, *i.e.*, microscopic cracks emerge and *B. cinerea* can easily penetrate the berries [15]. Furthermore, the accumulation of water between berries (slow drying of the berry surface) is reduced in loose bunches, which also restricts the appearance of bunch rot. For further statistical analysis, it was thus assumed that for grapevines with loose bunches, resistance to *B. cinerea* was not mainly influenced by the properties of cuticle, but the bunch architecture itself. The data from these grapevine genotypes were not considered in the regression analysis. In contrast, the *Pearson* correlation coefficients of the group of medium bunch compactness (Table 2) indicate a high negative relation between the impedance *Z_rel_* of CW and *B. cinerea* susceptibility. This relation is even 10% higher within the group of compact bunches. Hereby, the architecture of grape bunches is supposed as one major explanation. The correlation data in Table 2 indicate that the impedance of the cuticle is more correlated with *B. cinerea* resistance within the compact group than in the medium group. For the investigated genotypes, it could be assumed that genotypes with thick berry cuticles (high impedance) are more resistant than genotypes with thin cuticles (low impedance). In compact bunches, the berries touch each other (Figure 1), which causes injury to berry skin and subsequently promotes *B. cinerea* infection. It seems that the physical properties of the berry skin (thickness and permeability) in the group of compact bunches is more related to *B. cinerea* infection than in the group of medium bunches. This consideration indicates an overlay of bunch architecture and impedance of the cuticle and epicuticular waxes in relation to bunch rot.

**Figure 6:**
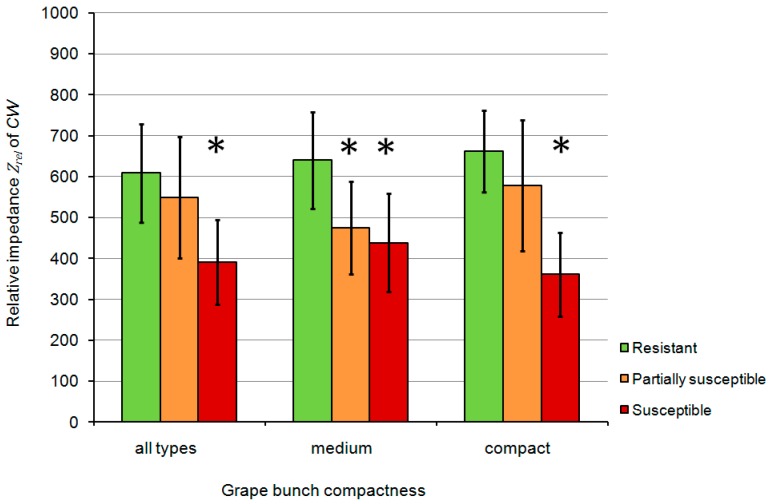
Mean relative impedance *Z_rel_* of CW of three different groups: resistant, partially susceptible and susceptible grapevines. The Duncan multiple range test (level of significance α = 0.05) was carried out to distinguish the groups statistically based on the mean impedance. Standard deviation is shown in the graphs. Stars indicate significant differences in comparison to the resistant group. CW: intact cuticle with epicuticular waxes.

To sum up, the *Z_rel_* of CW (*Z_rel_* of the cuticle with epicuticular waxes) showed the highest significant correlations. Thus, it was selected as the most promising indicator for the prediction of resistance of grape berries to *B. cinerea* using an ordinal regression analysis. However, the investigated genotypes were grouped on the basis of their corresponding *B. cinerea* susceptibility, *i.e.*, a resistant, partially susceptible, and susceptible group, and with regard to the level of bunch compactness (medium and compact). The mean impedance *Z_rel_* of CW with standard deviation was calculated for each group (Figure 6).

Significant differences of *Z_rel_* of CW were identified between the resistant and susceptible groups (Figure 6). No significant differences were observed between the average impedance of the resistant and partially susceptible group (all types of grape bunch compactness and compact bunches). Therefore, it could be helpful to investigate an enlarged set of cultivars. As a consequence, the distinction between susceptible and resistant grapevines is principally possible using the impedance value as a novel type of phenotypic data.

**Figure 7:**
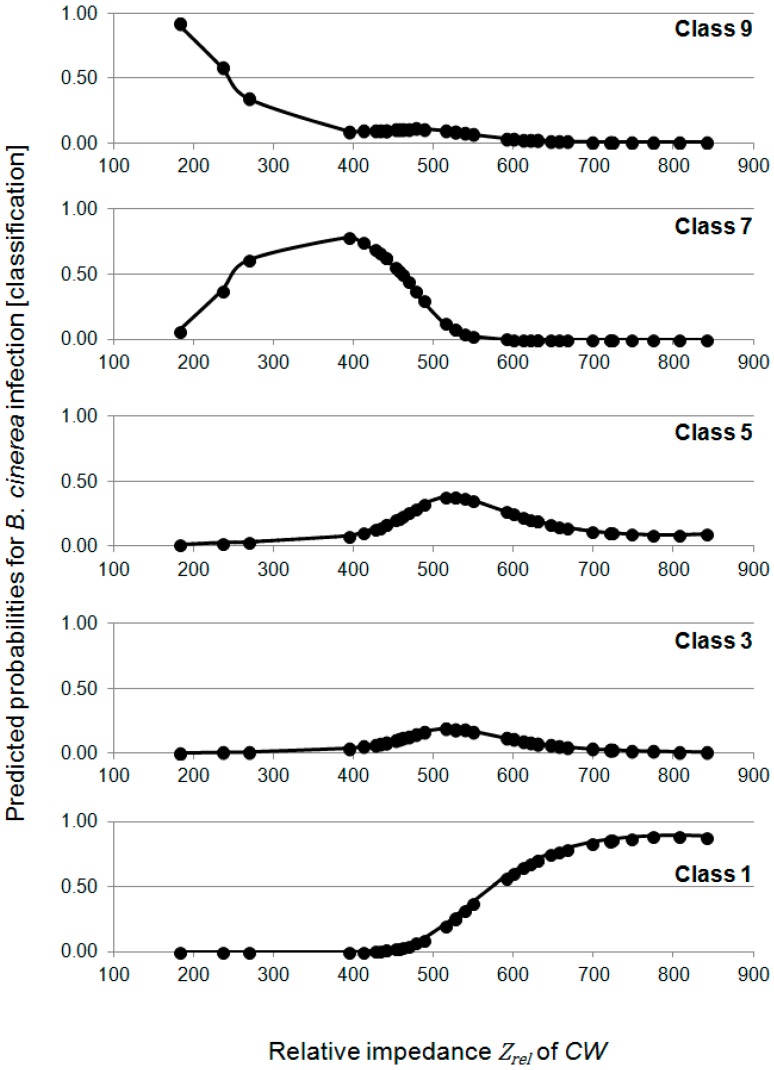
Prediction the probability of *B. cinerea* infection. Relative impedance *Z_rel_* of CW was applied in an ordinal logistic regression model. The data set including all genotypes (except genotypes with loose bunch compactness). Maximum Likelihood estimation was used and *R^2^_McFadden_* = 0.37 was calculated. CW: intact cuticle with epicuticular waxes.

Logistic regression analysis was carried out in order to predict the probability of *B. cinerea* infection by using the relative impedance *Z_rel_* of CW (Figure 7). 

In the regression analysis, an *R^2^*_McFadden_ of 0.37 was calculated. As visible in Figure 7, the classes 9, 7 and 1 of *B. cinerea* infection could be predicted from the simple measure *Z_rel_* of CW. The high *R^2^*_McFadden_ indicates that the impedance of CW is usable for the estimation of susceptibility of grapevines to *B. cinerea.* For further studies, it is an advantage that the impedance *Z_rel_* of CW is the most powerful phenotype, whereas the laborious removal of epicuticular waxes is not required. This is very important for common breeding questions because it enables the screening of hundreds of interesting genotypes in a short space of time by applying the simple to handle I-sensor.

**Figure 8:**
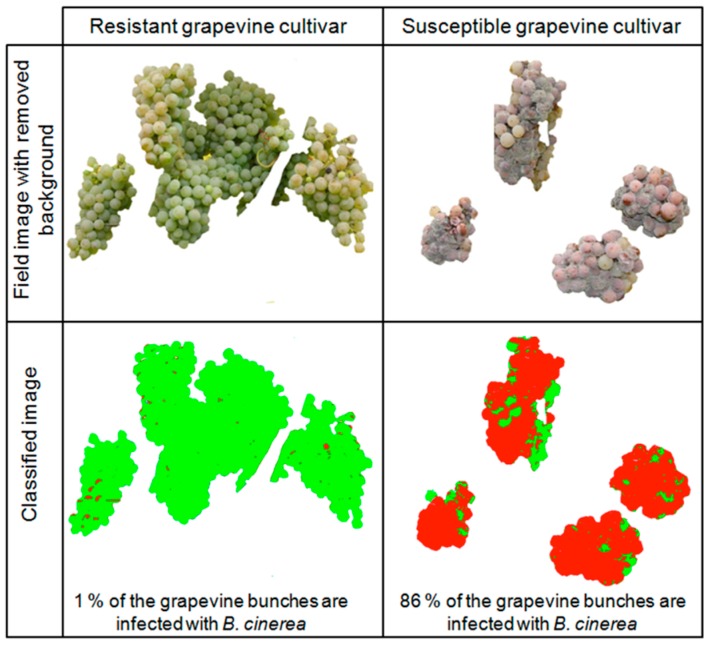
Exemplary image-based determination of *B. cinerea* infection on grapevine cultivars to improve reference evaluations of bunch rot in the field. The background of the field image was manually removed. Image segmentation into two phenotypic classes “healthy” (green) and “disease” (red) was performed by using Matlab^®^. The percentage amount of *B. cinerea* infection is quoted in the classified image.

For the generation of an improved regression model the set of investigated plants and further parameters should be included in the model, which may influence the susceptibility of grapevines to *B. cinerea*, e.g., the time of ripening or weather data (rainfall and temperature). In order to increase the objectivity of the model in the future, the *B. cinerea* infection of grapevine bunches could be determined with much more accuracy, e.g., by the application of objective, image-based phenotyping methods (an example is shown in Figure 8).

### 3.3. Impedance of the Berry Cuticle for QTL Analysis Applications

**Figure 9:**
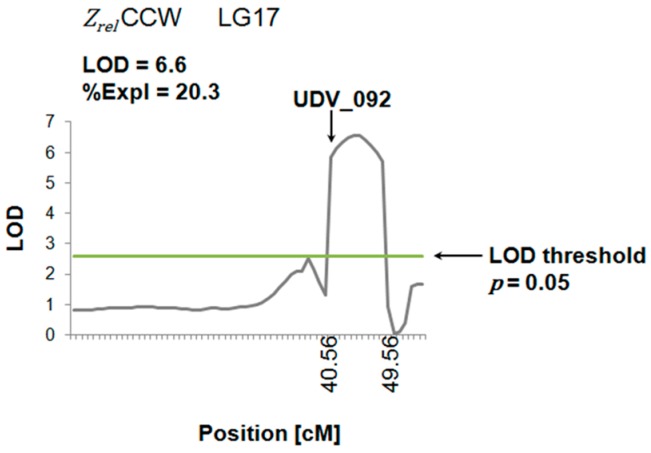
Preliminary QTL of the novel phenotypic trait in a F1 progeny (crossing of GF.GA.47-42 x 'Villard Blanc'). Genetic marker located within the detected QTL region is labeled with an arrow. The numbers on the x-axis indicate the position and confidence interval of the QTL region. LG = Linkage group; LOD = logarithm of the odds; %Expl = percentage of explained phenotypic variation. CCW: C (cuticle without epicuticular waxes) + CW (intact cuticle with epicuticular waxes)*.*

The development of genetic markers for grapevine breeding purposes requires the acquisition of phenotypic traits from mapping populations (=F1 progeny) and the computation of significant QTL regions with a high LOD (logarithm of the odds) value. In the present study, the novel phenotypic trait of grape berry, the impedance of the cuticle, was tested for its application in QTL studies. Therefore, the phenotypic data (*Z_rel_* of C, W, CW and CCW) received from the F1 progeny was analyzed. Preliminary QTLs were identified whose LOD value were above the significance level (*i.e.*, LOD threshold). One preliminary QTL on Linkage Group (LG) 17 is illustrated in Figure 9.

For further investigations including the development of genetic markers for grapevine breeding applications, repeated phenotpying of this population is required to narrow down the QTL regions and to normalize the phenotypic data regarding environmental influences. In contrast to laborious, traditional phenotyping methods [11], the sensor-based technique developed in this study will enable precise phenotyping of a large mapping population with increased throughput. With the background that the impedance seems to be a promising indicator for bunch rot resistance, genetic markers could be established facilitating the selection of grapevine breeding material with a high impedance of the cuticle and epicuticular waxes implicating a genetic improvement of the resistance of breeding material to bunch rot.

## 4. Conclusions

Until now, resistance donors of genetic loci to increase the resistance of grapevines to *B. cinerea* are widely unknown. In common grapevine-breeding programs, the selection of seedlings with convenient physical properties (loose bunch architecture and small berries) can be done to increase the resistance to *B. cinerea*. In the present study, the simple-to-handle I-sensor was established and related to that, a fast phenotyping method was developed to estimate the resistance of grape bunches to *B. cinerea*. The I-sensor enables the measurement of the impedance of the cuticle and its epicuticular waxes from grape berries implementing a novel phenotypic trait. The identification of a preliminary QTL within a F1 progeny of the crossing of GF.GA.47-42 x 'Villard Blanc' shows the feasibility of this novel trait for genetic analysis. However, correlation studies show a high negative correlation between the impedance of cuticle and epicuticular waxes and the susceptibility of berries to *B. cinerea.* The novel sensor-based phenotyping technique developed in this study can thus be used to characterize the cuticles of grape berries in an easy and fast manner. The level of impedance indicates the susceptibility of a grapevine cultivar to *B. cinerea* and thus provides a novel important determinant with regard to bunch rot resistance. This effect was observed for genotypes with medium and compact bunch architecture. For future work, an increased number of genotypes and further parameter should be considered to improve the regression model, e.g., the consideration of the time of ripening or weather data. The phenotyping of the mapping population by applying the I-sensor should also be an important task for future work in order to determine new genetic markers for marker-assisted selection (MAS) of grapevine seedlings with improved resistance to bunch rot. In addition, the I-sensor could be applied in common breeding programs or other viticultural experiments, e.g., in order to evaluate the effect of canopy reduction or other vineyard management strategies on the berry skin quality.

## Supplementary Files

Supplementary File 1

## References

[1] Jarvis W.R. (1977). Botryotinia and Botrytis Species: Taxonomy, Physiology, and Pathogenicity.

[2] Molitor D., Behr M., Fischer S., Hoffmann L., Evers D. (2011). Timing of cluster-zone leaf removal and its impact on canopy morphology, cluster structure and bunch rot susceptibility of grapes. J. Int. Sci. Vigne Vin..

[3] Broome J., English J., Marois J., Latorre B., Aviles J. (1995). Development of an infection model for botrytis bunch rot of grapes based on wetness duration and temperature. Phytopathology.

[4] Ellison P., Ash G., McDonald C. (1998). An expert system for the management of botrytis cinerea in australian vineyards. I. Development. Agric. Syst..

[5] Hed B., Ngugi H.K., Travis J.W. (2009). Relationship between cluster compactness and bunch rot in vignoles grapes. Plant Dis..

[6] Vail M., Marois J. (1991). Grape cluster architecture and the susceptibility of berries to botrytis cinerea. Phytopathology.

[7] Vail M., Wolpert J., Gubler W., Rademacher M. (1998). Effect of cluster tightness on botrytis bunch rot in six chardonnay clones. Plant Dis..

[8] Molitor D., Behr M., Hoffmann L., Evers D. (2012). Impact of grape cluster division on cluster morphology and bunch rot epidemic. Am. J. Enol. Vitic..

[9] Nair N.G., Allen R.N. (1993). Infection of grape flowers and berries by botrytis cinerea as a function of time and temperature. Mycol. Res..

[10] Deytieux-Belleau C., Geny L., Roudet J., Mayet V., Donèche B., Fermaud M. (2009). Grape berry skin features related to ontogenic resistance to botrytis cinerea. Eur. J. Plant Pathol..

[11] Gabler F.M., Smilanick J.L., Mansour M., Ramming D.W., Mackey B.E. (2003). Correlations of morphological, anatomical, and chemical features of grape berries with resistance to botrytis cinerea. Phytopathology.

[12] Kretschmer M., Kassemeyer H.H., Hahn M. (2007). Age-dependent grey mould susceptibility and tissue-specific defence gene activation of grapevine berry skins after infection by botrytis cinerea. J. Phytopathol..

[13] Nanni V., Schumacher J., Giacomelli L., Brazzale D., Sbolci L., Moser C., Tudzynski P., Baraldi E. (2014). Vvamp2, a grapevine flower-specific defensin capable of inhibiting botrytis cinerea growth: Insights into its mode of action. Plant Pathol..

[14] Commenil P., Brunet L., Audran J.-C. (1997). The development of the grape berry cuticle in relation to susceptibility to bunch rot disease. J. Exp. Bot..

[15] Becker T., Knoche M. (2012). Deposition, strain, and microcracking of the cuticle in developing ‘riesling’ grape berries. Vitis.

[16] Becker T., Knoche M. (2012). Water induces microcracks in the grape berry cuticle. Vitis.

[17] Schreiber L. (2010). Transport barriers made of cutin, suberin and associated waxes. Trends Plant Sci..

[18] Domínguez E., Heredia-Guerrero J.A., Heredia A. (2011). The biophysical design of plant cuticles: An overview. New Phytol..

[19] Benavente J., Ramos-Barrado J.R., Heredia A. (1998). A study of the electrical behaviour of isolated tomato cuticular membranes and cutin by impedance spectroscopy measurements. Colloids Surf. A Physicochem. Eng. Asp..

[20] Schreiber L. (2001). Effect of temperature on cuticular transpiration of isolated cuticular membranes and leaf discs. J. Exp. Bot..

[21] Schreiber L., Skrabs M., Hartmann K., Diamantopoulos P., Simanova E., Santrucek J. (2001). Effect of humidity on cuticular water permeability of isolated cuticular membranes and leaf disks. Planta.

[22] Zyprian E., Eibach R., Töpfer R. (2006). Eine neue genetische karte der weinrebe aus der kreuzung ‘‘GF.GA-47–42’’ x ‘‘villard blanc’’. Deutsches Weinbau Jahrbuch..

[23] Fechter I., Hausmann L., Zyprian E., Daum M., Holtgräwe D., Weisshaar B., Töpfer R. (2014). Qtl analysis of flowering time and ripening traits suggests an impact of a genomic region on linkage group 1 in vitis. Theor. Appl. Genet..

[24] Zyprian E., Ochßner I., Schwander F., Simon S., Bonow-Rex M., Moreno-Sanz P., Grando M.S., Wiedemann-Merdinoglu S., Merdinoglu D., Eibach R. Quantitative trait loci affecting resistance traits and ripening of grapevines in a genetic map based on single nucleotide polymorphisms and microsatellites.

[25] Lorenz D.H., Eichhorn K.W., Bleiholder H., Klose R., Meier U., Weber E. (1995). Growth stages of the grapevine: Phenological growth stages of the grapevine (vitis vinifera l. Ssp. Vinifera)-codes and descriptions according to the extended bbch scale. Aust. J. Grape Wine Res..

[26] OIV OIV publications: OIV descriptor list for grape varieties and Vitis species (2nd ed.). http://www.Oiv.int.

[27] Bühl A. (2008). Spss 16: Einführung in die Moderne Datenanalyse.

