# Supplementary material for: Impedance of the Grape Berry Cuticle as a Novel Phenotypic Trait to Estimate Resistance to Botrytis Cinerea
Source: Sensors (Basel). 2015 May 27;15(6):12498–512. doi: 10.3390/s150612498 (PMC4507599; doi:10.3390/s150612498)
Supplement: Supplementary File 1 [file sensors-15-12498-s001.pdf]

OPEN ACCESS

sensors

ISSN 1424-8220

www.mdpi.com/journal/sensors

Supplementary Information

Impedance of the Grape Berry Cuticle as a Novel Phenotypic Trait to Estimate Resistance to *Botrytis Cinerea*. *Sensors* 2015, *15*, 12498-12512

Katja Herzog *, Rolf Wind and Reinhard Töpfer

Julius Kühn-Institute-Federal Research Centre for Cultivated Plants, Institute for Grapevine Breeding Geilweilerhof, Siebeldingen 76833, Germany; E-Mails: rolf.wind@kdwelt.de (R.W.); reinhard.toepfer@jki.bund.de (R.T.)

* Author to whom correspondence should be addressed; E-Mail: Katja.herzog@jki.bund.de; Tel.: +49-6345-41-114; Fax: +49-6345-91-9050.

Table S1. Investigated grapevine material. The column 'genotypes' indicate the names of the cultivars or breeding material, different years of investigation or divergent viticultural cultivation is declared. The relative impedance values, the class of susceptibility of investigated grapevines to *B. cinerea* and the compactness of grapevine bunches are presented. 1 = no and 9 = high *B. cinerea* susceptibility; 1 = loose and 9 = compact bunch architecture. *Zrel*: relative Impedance; C: cuticle without epicuticular waxes; CW: intact cuticle with epicuticular waxes; W: epicuticular wax; CCW: C + CW.

Genotypes	*Zrel* CW	*Zrel* C	*Zrel* W	*Zrel* CCW	*B. cinerea* Susceptibility	Bunch Compactness
13-37-27 *[1]	697	226	472	923	1	7
13-39-07 *[1]	433	172	261	605	7	3
13-39-09 *[1]	655	255	400	910	3	7
13-39-11 *[1]	666	219	447	885	5	7
13-41-01 *[1]	486	206	280	692	3	7
13-41-17 *[1]	629	236	393	864	1	7
13-42-01 *[1]	493	238	255	731	1	3
13-42-19 *[1]	354	145	209	500	7	3
13-44-19 *[1]	645	258	387	903	1	3
1993-022-006 *[2]	645	223	422	868	1	5
Bacchus	393	69	324	462	7	7
Beyer 2 *[3]	620	214	406	834	1	7
Chardonnay	431	217	214	648	7	7
Chardonnay (MPTS *[4])	548	234	314	781	1	5
De Chaunac *[3] (2013)	840	208	633	1048	1	5
De Chaunac *[3] (2014)	719	237	482	956	1	5
Freiburg 868-59 *[3]	476	152	325	628	5	7

Table S1. *Cont.*

Genotypes	*Zrel* CW	*Zrel* C	*Zrel* W	*Zrel* CCW	*B. cinerea* Susceptibility	Bunch Compactness
Geilweilerhof C- 41- 44 *[3]	546	206	339	752	1	3
Gewürztraminer	439	123	316	561	5	7
Kentville White 94-2 *[3]	746	143	603	889	1	5
Kerner	513	111	402	624	7	5
Morio Muskat (2013)	268	167	101	434	7	7
Morio Muskat (2014)	291	106	185	397	7	7
Müller-Thurgau	439	115	324	554	7	5
Muskateller	235	82	153	316	9	5
Optima	181	37	143	218	9	7
Pinot Blanc	410	97	313	506	7	7
Pinot Noir	460	150	309	610	7	7
Reflex *3	348	133	215	482	1	3
Regent	425	150	275	576	5	5
Regent	467	169	298	636	7	5
Regent (MPTS *[4])	484	173	312	657	3	3
Riesling	589	236	353	825	5	7
Riesling (MPTS *[4])	598	272	326	870	1	5
Seibel 8616 *[3]	722	199	523	921	5	7
Seibel 9249 *[3]	455	90	365	546	9	7
Seibel 10868 *[3]	525	156	369	681	1	5
Seibel 11259-16 *[3]	537	209	328	746	9	5
Seibel 12084 *[3]	438	85	352	523	1	3
Seibel 13047 *[3]	610	157	452	767	1	5
Seibel 13770 *[3]	451	173	278	624	3	5
Seibel 182 *[3] (2013)	773	335	438	1108	1	7
Seibel 182 *[3] (2014)	806	290	516	1095	1	7
Seibel 5318 *[3]	525	210	316	735	5	5
Vidal Blanc (2013)	582	259	323	841	1	3
Vidal Blanc (2014)	614	276	337	890	3	3
Vineland 67155 *[3]	628	297	331	926	1	7
Zala Gyoengye *[3]	719	289	430	1007	1	5

*[1] Genotypes from the population GF.GA-47-42 x 'Villard Blanc'; *[2] Breeding material; *[3] Grapevines from the genetic repository; *[4] Minimal Pruning of Trellis System–it is a new cultivation method resulting in a looser grape bunch compactness.

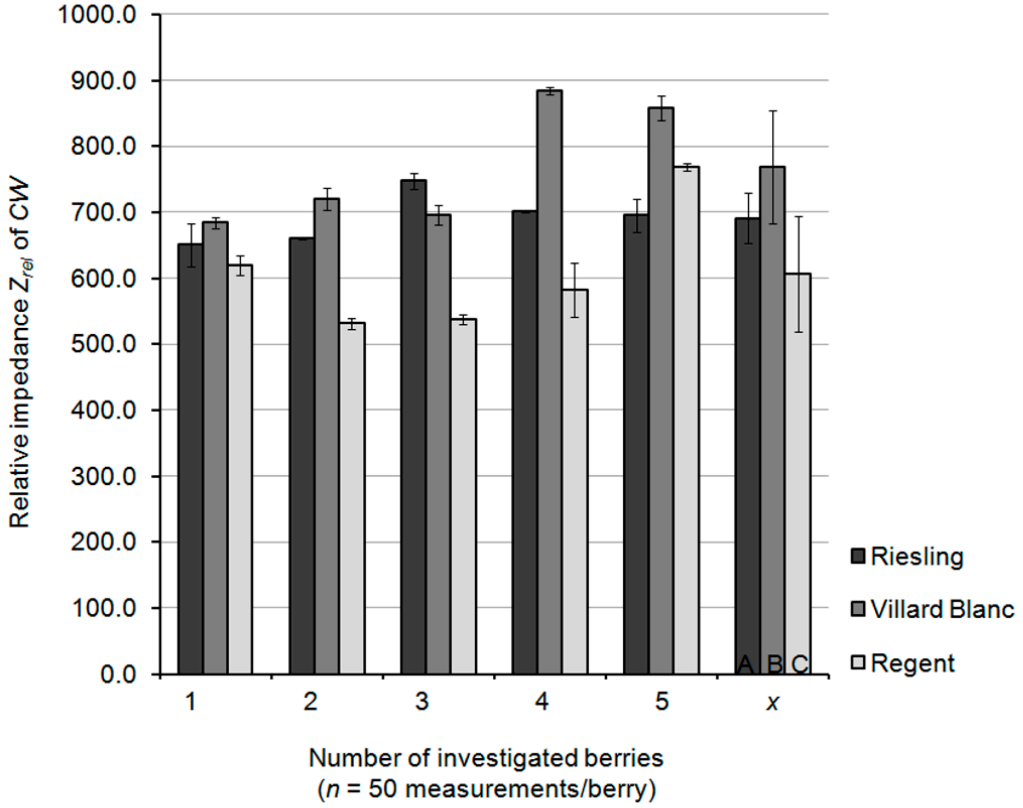

Figure S1. Investigation of the repeatability of impedance measurements. Z_{rel} of CW was measured 50 times per berry at one puncture site and five berries per cultivar. x represents the mean Z_{rel} for a cultivar. The standard deviation is illustrated in the bars. The *Duncan* multiple range test ($p = 0.05$) was conducted–different letters indicate significant differences.

© 2015 by the authors; licensee MDPI, Basel, Switzerland. This article is an open access article distributed under the terms and conditions of the Creative Commons Attribution license (http://creativecommons.org/licenses/by/4.0/).
